# Effects of Posaconazole on Tacrolimus Population Pharmacokinetics and Initial Dose in Children With Crohn’s Disease Undergoing Hematopoietic Stem Cell Transplantation

**DOI:** 10.3389/fphar.2022.758524

**Published:** 2022-04-13

**Authors:** Xiao Chen, Dongdong Wang, Feng Zheng, Lin Zhu, Yidie Huang, Yiqing Zhu, Ying Huang, Hong Xu, Zhiping Li

**Affiliations:** ^1^ Department of Pharmacy, Children’s Hospital of Fudan University, Shanghai, China; ^2^ Department of Gastroenterology, Children’s Hospital of Fudan University, Shanghai, China; ^3^ Department of Nephrology, Children’s Hospital of Fudan University, National Children’s Medical Center, Shanghai, China

**Keywords:** posaconazole, tacrolimus, population pharmacokinetics, Crohn’s disease, hematopoietic stem cell transplantation

## Abstract

The present study explored the effects of posaconazole on tacrolimus population pharmacokinetics (PPK) in children with Crohn’s disease (CD) undergoing hematopoietic stem cell transplantation (HSCT). Tacrolimus concentrations, physiological and biochemical factors, and concomitant medications from 51 CD children undergoing HSCT were used to establish a PPK model based on a nonlinear mixed-effect model. Steady-state concentrations of tacrolimus for children weighing less than 20 kg treated with different dose regimens were simulated by the Monte Carlo method. Weight and concomitant medications were included as covariates. At the same weight, the relative tacrolimus clearance was 1:0.43 in children without or with posaconazole. Compared to children not receiving posaconazole, the simulated tacrolimus steady-state concentrations at different doses for different body weights were all higher in children receiving posaconazole (*p* < 0.01). Furthermore, in children not receiving posaconazole, the dosage regimen with the best probability of achieving the target concentration was 0.6 mg/kg/day for children weighing 5–8.2 kg and 0.5 mg/kg/day for children weighing 8.2–20 kg, while for children receiving posaconazole, the best probability of reaching the target concentration of tacrolimus was a dosage regimen of 0.5 mg/kg/day for children weighing 5–20 kg. In conclusion, the PPK for tacrolimus was determined in children with CD undergoing HSCT for the first time. Co-treatment with posaconazole significantly increased tacrolimus concentrations, and we recommend a specific initial dose regimen for tacrolimus.

## Introduction

Crohn’s disease (CD) is an inflammatory bowel disease of unknown causes that may occur anywhere along the gastrointestinal tract (Torres et al., 2017). Uncontrolled inflammation results in long-term complications, including enteric fistulae, fibrotic strictures, and intestinal neoplasia (Cushing and Higgins, 2021). These symptoms may be observed in childhood as an outcome of undergoing surgical operation and may ultimately develop into short gut syndrome (Ruiz et al., 2020). The treatment outcomes for CD are not entirely satisfactory (Ruiz et al., 2020). For example, prescription medication treatments require prolonged treatment, and some patients become refractory. In addition, recurrent surgeries force patients to search for alternative treatments, including hematopoietic stem cell transplantation (HSCT) (Ruiz et al., 2020).

It has been reported that HSCT significantly improves the quality of life of CD patients, and 50% of CD patients who underwent HSCT achieved complete healing of the mucosa and clinical remission (Lindsay et al., 2017; Lopez-Garcia et al., 2017; Ruiz et al., 2020). However, for HSCT patients, long-term tacrolimus treatment is necessary to prevent rejection (Gao and Ma, 2019; Ishiwata et al., 2020; Soskind et al., 2020; Zhou et al., 2020). Similarly, posaconazole is also needed for the prevention of invasive fungal disease (IFD) in HSCT patients (Busca et al., 2016; Zhang et al., 2017). Posaconazole is a well-known inhibitor of CYP3A4, and its effects on tacrolimus have been described previously. Currently, it has been reported that posaconazole may influence tacrolimus concentrations in adult lung transplant recipients (Chanoine et al., 2020). However, CD as an intestinal disease, which may influence the *in vivo* bioavailability of the drug and its effects on tacrolimus concentrations, also needs further study. Moreover, the effects of posaconazole on tacrolimus concentrations in CD patients undergoing HSCT, especially children, remain unknown.

Therefore, the present study aimed to explore the effects of posaconazole on tacrolimus population pharmacokinetics (PPK) in CD children undergoing HSCT.

## Methods

### Patient Information

Pediatric patients diagnosed with CD and undergoing HSCT treated with tacrolimus from October 2017 to December 2020 at the Children’s Hospital of Fudan University (Shanghai, China) were collected, retrospectively. Partial basic clinical data of children were collected from a previous study (Wang et al., 2020). Given the retrospective nature of the study, the Ethics Committee of the Children’s Hospital of Fudan University (Ethical code: [2019] 020) approved the study and waived the need for written informed consent. The oral administration of tacrolimus was 0.33–2 mg/day for the initial dosage, and the tacrolimus dosage was later adjusted based on the clinical efficacy and adverse events experienced by patients and its trough concentration in therapeutic drug monitoring (TDM), where the concentration collection point of tacrolimus was before the next tacrolimus administration in order to get the tacrolimus trough concentration. The management of posaconazole was performed by physicians based on experience and actual clinical conditions of patients. In this study, posaconazole was analyzed as a categorical covariable. Tacrolimus concentrations were tested by the Emit^®^ 2000 Tacrolimus Assay (Siemens Healthcare Diagnostics Inc., Newark, NJ, United States) with a range of 2.0–30 mg/ml.

### PPK Model

The tacrolimus PPK model was established using nonlinear mixed-effects modeling software, NONMEM (edition 7, ICON Development Solutions, Ellicott City, MD, United States) and a first-order conditional estimation with interaction (FOCE-I) method. All the tacrolimus concentrations in this study were trough concentrations, and a one-compartment model was used for the present study. Apparent oral clearance (CL/F), volume of distribution (V/F), and the absorption rate constant (Ka) fixed at 4.48/h (Yang et al., 2015; Wang D.-D. et al., 2019) were included in the pharmacokinetic parameters.

### Random-Effects Model

Equation 1 was used to estimate between-subject variability:

$$P_{i}=\mathrm{TV}\left(P\right)\times \exp\left(\eta_{i}\right),$$ (1)

where P_i_ was the individual parameter value; TV(P), the typical individual parameter value; and η_i_, the symmetrical distribution, a random term with zero mean and variance omega^2 (ω^2^).

Equation 2 was used to estimate random residual variability:

$$O_{i}=IP_{i}\times \left(1+\varepsilon_{1}\right)+\varepsilon_{2},$$ (2)

where O_i_ was the observed concentration; IP_i_, the individual predicted concentration; and ε_1_ and ε_2_, symmetrical distribution, a random term with zero mean and variance sigma^2 (σ^2^).

### Covariate Model

Equation 3 was used to estimate pharmacokinetic parameters and weight:

$$B_{i}=B_{\mathrm{std}}\times {\left(W_{i}/W_{\mathrm{std}}\right)}^{\mathrm{POW}},$$ (3)

where B_i_ was the *i*th individual parameter; B_std_, the typical parameter; W_i_, the *i*th individual weight; W_std_, the standard weight of 70 kg. POW; and the allometric coefficient was 0.75 for the CL/F and 1 for the V/F (Anderson and Holford, 2008).

Equations 4, and 5 were used to estimate the pharmacokinetic parameters and continuous covariates or categorical covariates:

$$P_{i}=\mathrm{TV}\left(P\right)\times {\left(\mathrm{Co}v_{i}\mathrm{/Co}v_{\mathrm{median}}\right)}^{\theta },$$ (4)

$$P_{i}=\mathrm{TV}\left(P\right)\times \left(\mathrm{1+\theta}\times \mathrm{Co}v_{i}\right),$$ (5)

where P_i_ was the individual parameter value; TV(P), the typical individual parameter value; θ, the parameter to be estimated; Cov_i_, the covariate of the *i*th individual; and Cov_median_, the population median for the covariate.

Demographic data (sex, age, and weight), clinical and biochemical parameters (albumin, alanine transaminase, aspartate transaminase, creatinine, urea, total protein, total bile acid, direct bilirubin, total bilirubin, hematocrit, hemoglobin, mean corpuscular hemoglobin, and mean corpuscular hemoglobin concentration), and co-medications (glucocorticoids, mycophenolic acid, omeprazole, and posaconazole) were collected. These were also analyzed as potential covariates. The covariates were screened in a stepwise fashion with forward inclusion and backward elimination, and the effect of each variable on the parameters was investigated using the likelihood ratio (Cai et al., 2020). Objective function value (OFV) changes were calculated as the covariate inclusion criteria. A decrease of OFV >6.63 (*p* < 0.01) defined the inclusion standard, and an increase of OFV >10.8 (*p* < 0.001) defined the exclusion standard.

### Model Evaluation

Model evaluation was carried out by observations *vs.* population predictions, observations *vs*. individual predictions, conditional weighted residuals (CWRES) *vs*. population predictions, CWRES *vs*. time after the start of therapy, and visual predictive check (VPC) of the model and individual plots. In addition, 1000 bootstraps with different random sampling were used to evaluate model stability.

### Simulation

The steady-state concentrations of tacrolimus were simulated for the presence or absence of concomitant treatment with posaconazole. In every scenario, 1000 virtual CD children undergoing HSCT were simulated in four body weight groups (5, 10, 15, and 20 kg) for eight doses (0.1 mg/kg/day, 0.2 mg/day, 0.3 mg/day, 0.4 mg/ day, 0.5 mg/day, 0.6 mg/day, 0.7 mg/day, and 0.8 mg/day). The daily dose was divided evenly into two doses. Tacrolimus concentrations were within the range of 5–20 ng/ml.

## Results

### Patient Information

Fifty-one CD children undergoing HSCT were included in the present study, 32 boys and 19 girls, aged 0.27–7.58 years. In total, 424 tacrolimus concentrations were included in the analysis, and the average concentrations evaluated were eight per patient. Demographic data of patients and drug combination are shown in Table 1 (Demographic data between who received posaconazole and who did not are shown in Supplementary Table S1).

**TABLE 1:** Demographic data of patients and drug combination (*n* = 51).

Characteristic	Mean ± SD	Median (Range)
Gender (boys/girls)	32/19	—
Age (years)	1.86 ± 1.38	1.36 (0.27–7.58)
Weight (kg)	9.85 ± 3.41	9.50 (3.70–20.60)
Albumin (g/L)	34.62 ± 3.61	34.40 (27.50–43.30)
Alanine transaminase (IU/L)	57.31 ± 124.13	25.60 (7.70–789.40)
Aspartate transaminase (IU/L)	57.07 ± 87.63	37.00 (12.50–628.20)
Creatinine (μmol/L)	17.55 ± 3.80	17.00 (11.00–28.00)
Urea (mmol/L)	2.92 ± 1.27	2.90 (0.70–6.90)
Total protein (g/L)	59.43 ± 6.21	58.50 (48.10–72.20)
Total bile acid (μmol/L)	7.47 ± 7.18	5.20 (0.90–33.90)
Direct bilirubin (μmol/L)	3.45 ± 6.99	2.30 (0.80–51.80)
Total bilibrubin (μmol/L)	8.62 ± 11.43	6.60 (2.90–85.60)
Hematocrit (%)	30.00 ± 3.89	30.10 (21.40–42.50)
Hemoglobin (g/L)	95.29 ± 13.90	95.00 (66.00–147.00)
Mean corpuscular hemoglobin (pg)	24.95 ± 2.79	25.10 (18.30–29.90)
Mean corpuscular hemoglobin concentration (g/L)	317.31 ± 17.70	318.00 (280.00–348.00)
Number of co-medications		
Glucocorticoids	40	
Mycophenolic acid	26	
Omeprazole	41	
Posaconazole	12	

### Modeling and Evaluation

The covariates were included based on forward inclusion and backward elimination (the stepwise fashion with forward inclusion and backward elimination is shown in Supplementary Table S2). The final model was as follows:

$$\mathrm{CL/F=19}\mathrm{.8}\times {\left(\mathrm{WT/70}\right)}^{0.75}\times \left(1-0.57\times \mathrm{POS}\right),$$ (6)

V/F=11300×(WT/70), (7)

where WT represented weight and POS represented posaconazole. When a patient was treated with posaconazole, the POS value was set to 1; otherwise, the POS value was set to 0.

**FIGURE 1:**
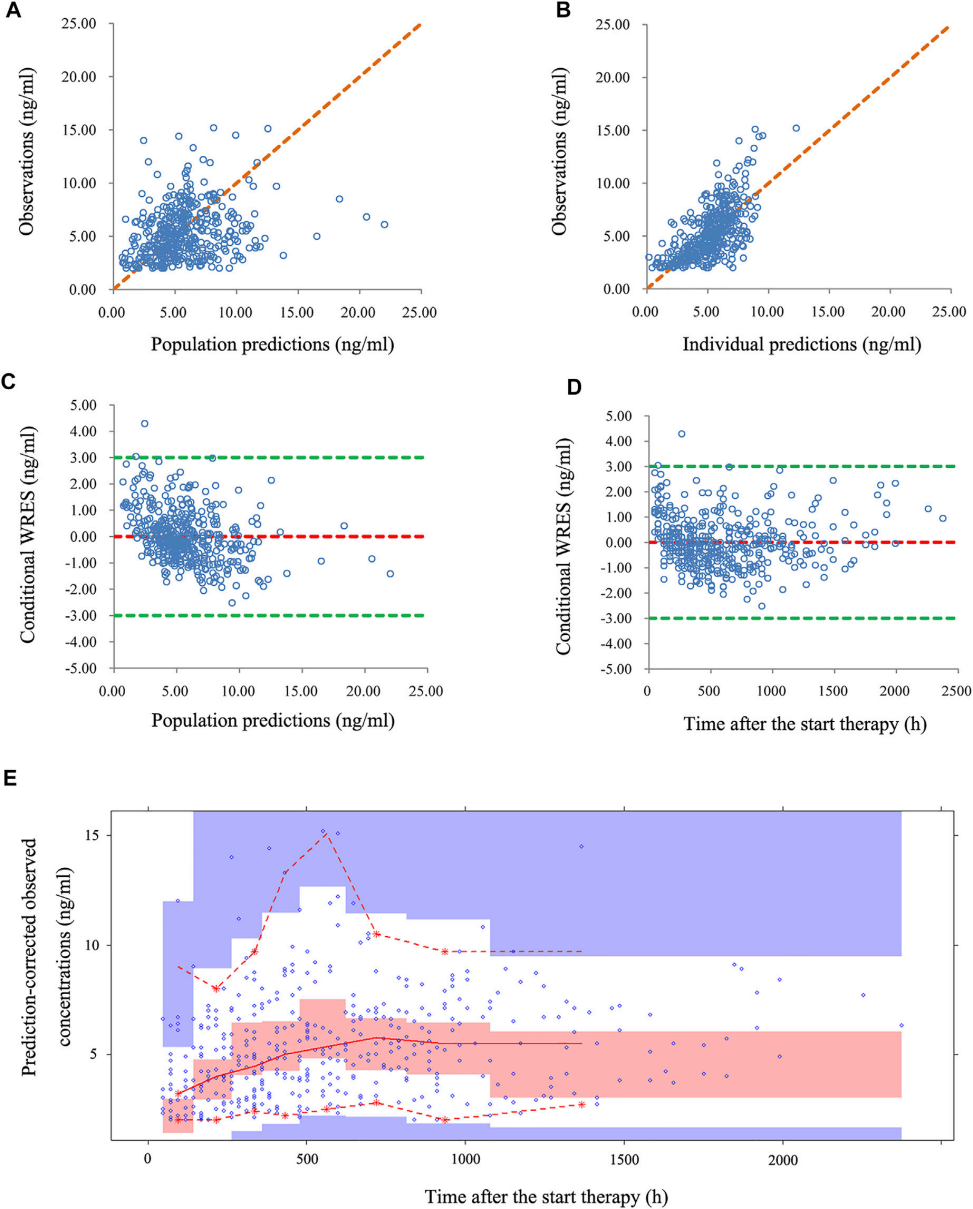
Model evaluation. **(A)** Observations *vs.* population predictions. **(B)** Observations *vs*. individual predictions. **(C)** Conditional weighted residuals (CWRES) *vs*. population predictions. **(D)** CWRES *vs*. time after the start of therapy. **(E)** Visual predictive check (VPC) of the model. The middle solid line represents the median of the prediction-corrected concentrations. The lower and upper dashed lines are the 2.5th and 97.5th percentiles of the prediction-corrected concentrations, respectively.

**FIGURE 2:**
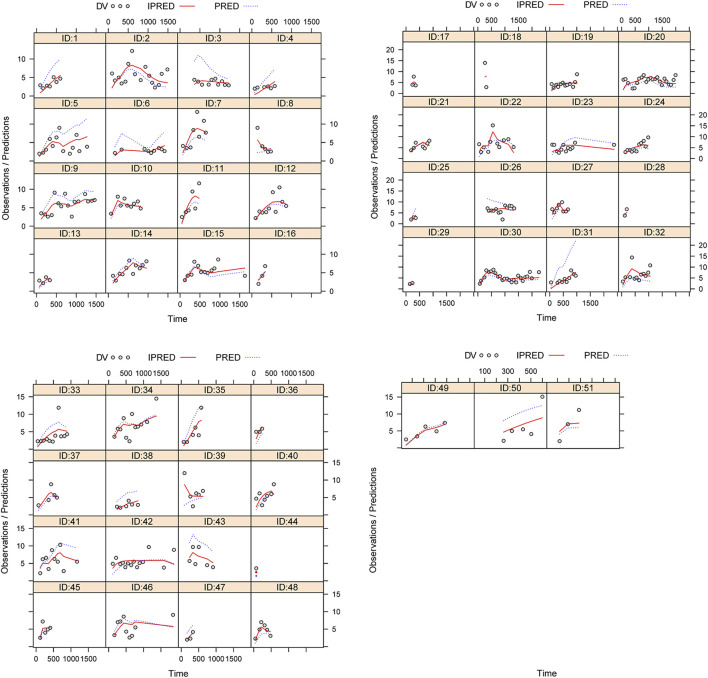
Individual plot ID: patient ID number. DV: measured concentration value. IPRED: individual predictive value. PRED: population predictive value.

Figure 1 illustrated the model evaluation. Figure 1A compared the observations with population predictions, and conversely, Figure 1B compared observations with individual predictions (observations *vs.* individual predictions in children with CD undergoing HSCT who received posaconazole and those who did not are shown in Supplementary Figure S1). Figure 1C showed the CWRES *vs*. population predictions, and Figure 1D showed the CWRES *vs*. time after the start of therapy. Figure 1E was the VPC of the model, in which most of the observed concentrations were within the 95% prediction intervals of the simulation data, indicating that the prediction-corrected concentrations were well-predicted by the final model. Table 2 summarizes the parameter estimates of the final model and bootstrap validation. The median values of the bootstraps were similar to the respective parameter values of the final model, and the bias absolute values were less than 5%, indicating that the model was reliable and accurate. In addition, individual plots are shown in Figure 2; from the perspective of sparse clinical data, the present model demonstrated acceptable predictability.

**TABLE 2:** Parameter estimates of the final model and bootstrap validation.

Parameter	Estimate	SE (%)	Bootstrap		Bias (%)
			Median	95% confidence interval	
CL/F (L/h)	19.8	7.0	19.8	[16.5, 23.7]	0
V/F (10^2^L)	113	13.9	114	[85, 148]	0.885
Ka (h^−1^)	4.48 (fixed)	—	—	—	—
θ_POS_	−0.57	12.1	−0.58	[−0.73, −0.26]	1.754
ω_CL/F_	0.349	15.8	0.339	[0.131, 0.539]	−2.865
ω_V/F_	0.859	14.7	0.831	[0.501, 1.077]	−3.260
σ_1_	0.259	11.8	0.258	[0.169, 0.314]	−0.386
σ_2_	1.353	13.2	1.356	[0.966, 1.723]	0.222

The 95% confidential interval was displayed as the 2.5^th^ and 97.5th percentiles of bootstrap estimates. CL/F, apparent oral clearance (L/h); V/F, apparent volume of distribution (L); Ka, absorption rate constant (h^−1^); θ_POS_, was the coefficient of the posaconazole; ω_CL/F_, interindividual variability of CL/F; ω_V/F_, interindividual variability of V/F; σ_1_, residual variability, proportional error; σ_2_, residual variability, additive error; bias, prediction error, bias = (median-estimate)/estimate×100%.

### Effects of Posaconazole on Tacrolimus in CD Children Undergoing HSCT

**FIGURE 3:**
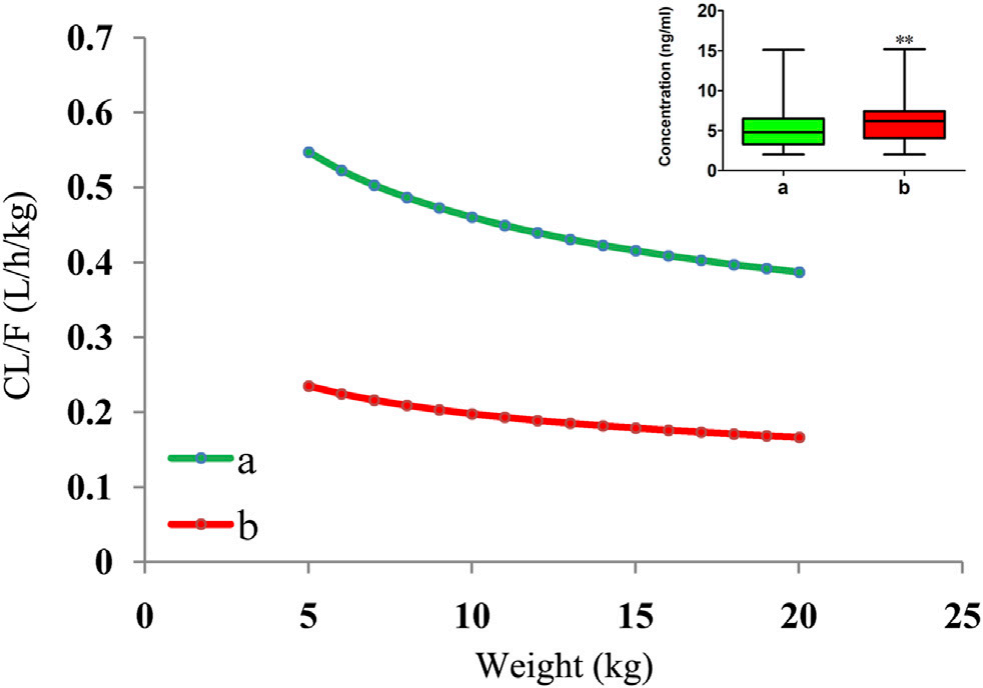
Tacrolimus CL/F in CD children undergoing HSCT. a: without posaconazole. b: with posaconazole. ^**^ *p <* 0.01 *vs.* children without posaconazole (measured tacrolimus concentrations).

**FIGURE 4:**
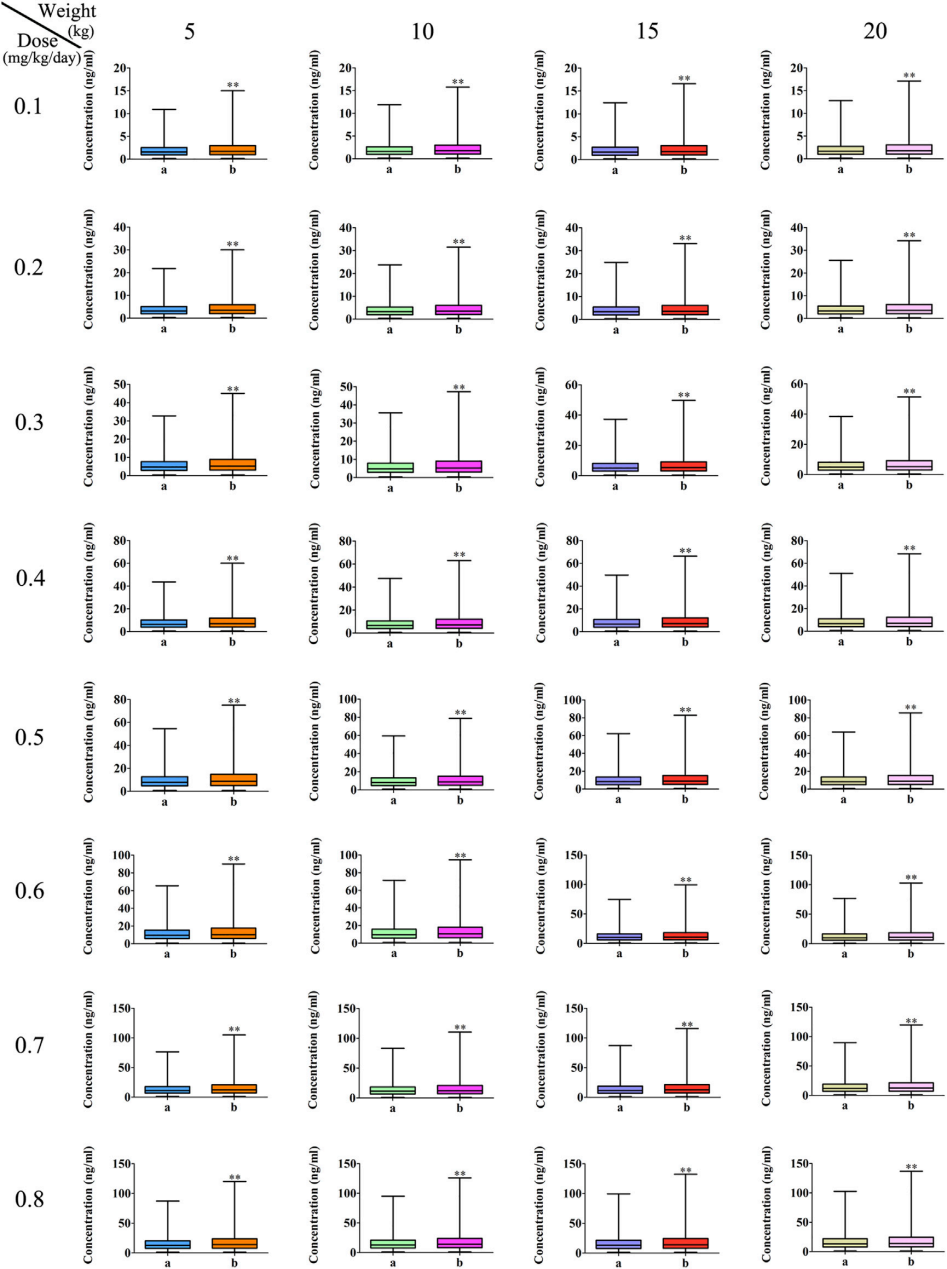
Effects of posaconazole on tacrolimus concentrations. a: without posaconazole. b: with posaconazole. *^*^ *p <* 0.01 *vs.* children without posaconazole.

The measured tacrolimus concentrations, compared to children without posaconazole, were all higher in children receiving posaconazole (*p* < 0.01), and for the CL/F of tacrolimus in CD children undergoing HSCT of the same weight, the relative value of tacrolimus clearance was 1:0.43 in children without or with posaconazole, as shown in Figure 3. However, concentration differences had not been corrected for the effects of dose and body weight. Thus, we further simulated tacrolimus concentrations for different body weights (range, 5–20 kg) and different dosage regimens (0.1–0.8 mg/kg/day), and the results indicated that at the same body weight and same dose, tacrolimus steady-state concentrations in children with posaconazole were, indeed, higher than those in children not receiving posaconazole (*p* < 0.01), as shown in Figure 4. The abovementioned results suggests that posaconazole significantly increased tacrolimus concentrations in CD children undergoing HSCT, and attention should be paid to the adjustment of drug dose to prevent the occurrence of toxicity when the two drugs are combined.

**FIGURE 5:**
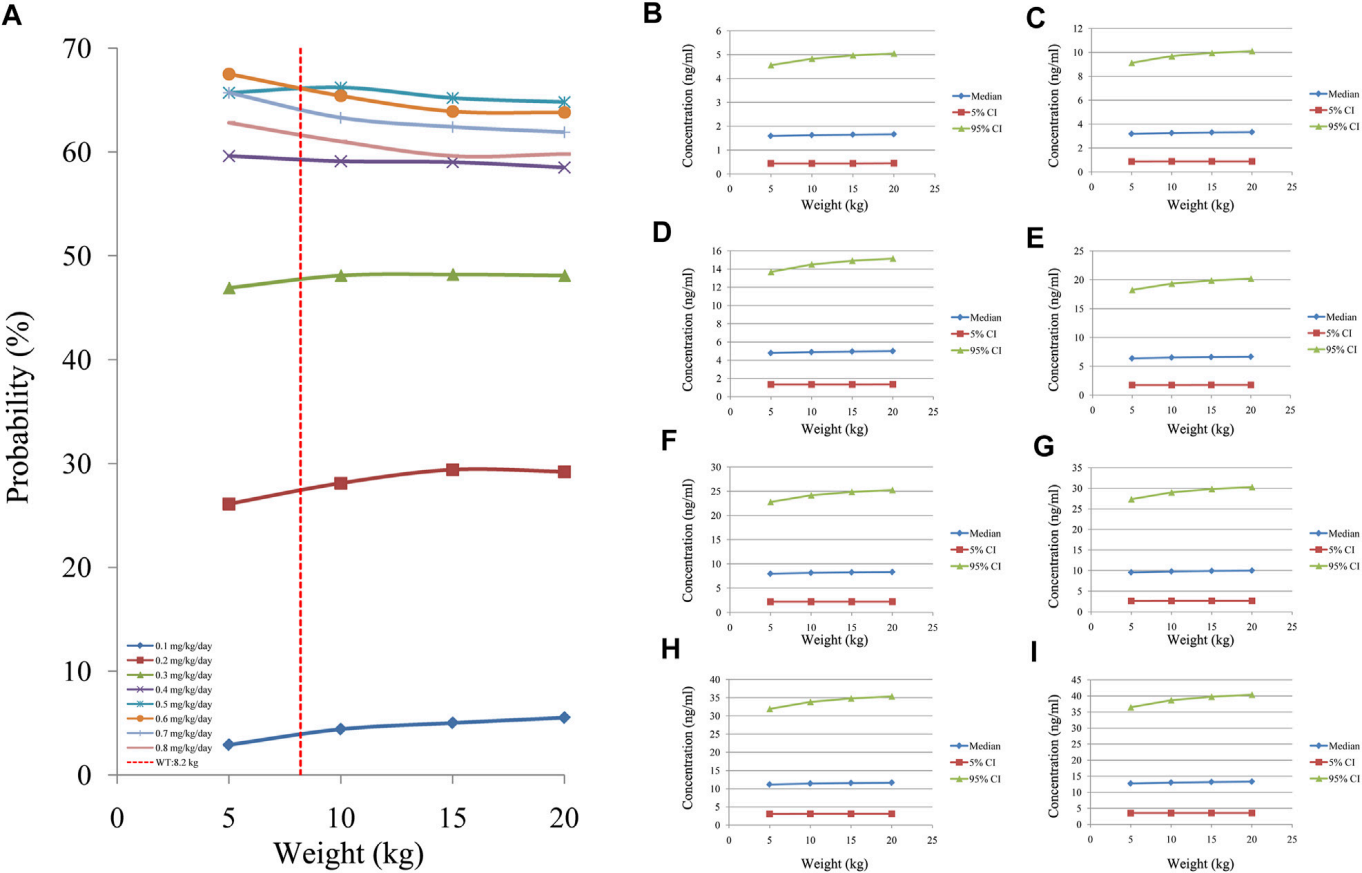
Probability of tacrolimus concentrations in CD children undergoing HSCT without posaconazole. **(A)** Probability of reaching tacrolimus concentrations (5–20 ng/ml). **(B)** 0.1 mg/kg/day tacrolimus-simulated concentrations. **(C)** 0.2 mg/kg/day tacrolimus-simulated concentrations. **(D)** 0.3 mg/kg/day tacrolimus-simulated concentrations. **(E)** 0.4 mg/kg/day tacrolimus-simulated concentrations. **(F)** 0.5 mg/kg/day tacrolimus-simulated concentrations. **(G)** 0.6 mg/kg/day tacrolimus-simulated concentrations. **(H)** 0.7 mg/kg/day tacrolimus-simulated concentrations. **(I)** 0.8 mg/kg/day tacrolimus-simulated concentrations. Median, 5% CI; 95% CI were median value, 5% and 95% confidence interval of 1000 times simulation.

**FIGURE 6:**
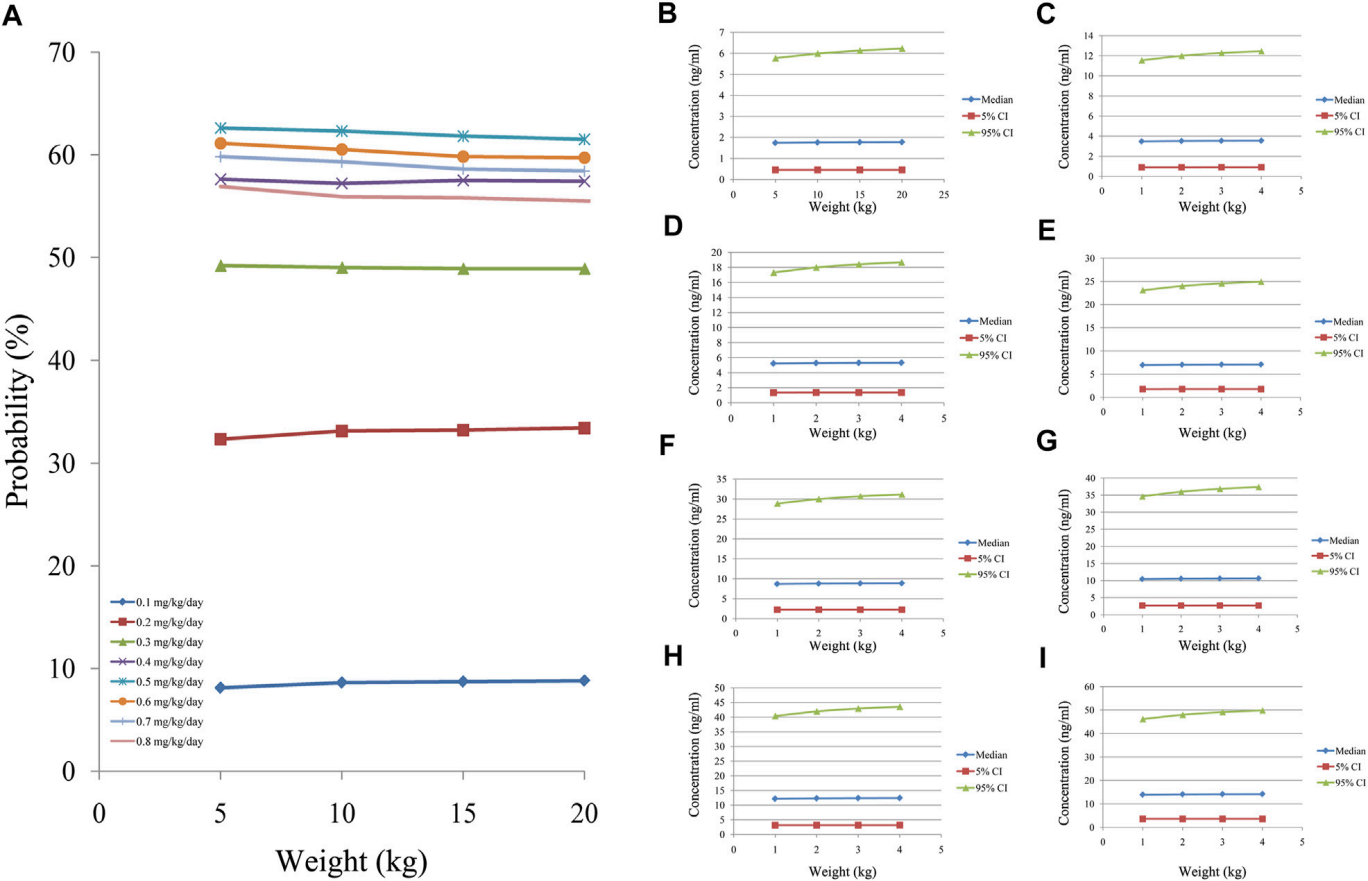
Probability of tacrolimus concentrations in CD children undergoing HSCT with posaconazole. **(A)** Probability of reaching tacrolimus concentrations (5–20 ng/ml). **(B)** 0.1 mg/kg/day tacrolimus-simulated concentrations. **(C)** 0.2 mg/kg/day tacrolimus-simulated concentrations. **(D)** 0.3 mg/kg/day tacrolimus-simulated concentrations. **(E)** 0.4 mg/kg/day tacrolimus-simulated concentrations. **(F)** 0.5 mg/kg/day tacrolimus-simulated concentrations. **(G)** 0.6 mg/kg/day tacrolimus-simulated concentrations. **(H)** 0.7 mg/kg/day tacrolimus-simulated concentrations. **(I)** 0.8 mg/kg/day tacrolimus-simulated concentrations. Median, 5% CI; 95% CI were median value, 5% and 95% confidence interval of 1000 times simulation.

Figure 5 showed the tacrolimus concentrations at different doses for different body weights in CD children undergoing HSCT without posaconazole treatment. As shown in Figure 5A, the best probabilities for reaching the target concentrations were 0.6 mg/kg/day for children weighing 5–8.2 kg and 0.5 mg/kg/day for children weighing 8.2–20 kg. Figures 5B–I show the tacrolimus concentration ranges of different dosage regimens, respectively. Figure 6 shows the tacrolimus concentrations at different doses for different body weights in CD children undergoing HSCT and treated with posaconazole. The best probabilities of reaching the target were tacrolimus concentrations of 0.5 mg/kg/day for children weighing 5–20 kg. Figures 6B–I show tacrolimus concentration ranges of different dosage regimens, respectively.

## Discussion

The rationale for the treatment of CD by HSCT originates from a series of case reports about coincidental CD remission subsequent to HSCT carried out for a standard neoplastic or hematologic indication (Ditschkowski et al., 2003). The first report describing HSCT performed for CD treatment was in 2003, and subsequently, a series of studies investigating this indication in CD were launched in many countries. If the therapeutic approach was evaluated based on the traditional prescription drug study criteria, HSCT promoted significant endoscopic mucosal healing and increased the quality of life of CD patients (Lindsay et al., 2017). Based on data collected by the European Society for Blood and Marrow Transplantation Registry, CD is currently the third most common autoimmune disease treated using HSCT (Snowden et al., 2018), which reveals the potential value of HSCT in the treatment of CD.

Post-transplant immunologic complications, such as acute and chronic graft-versus-host disease (GVHD), are obstacles to successful transplantation outcomes (Wang et al., 2020). Thus, it is vital to prevent the risk of developing GVHD. Tacrolimus is currently the first-line drug used for the prevention of GVHD following HSCT and has played an important role in addressing this urgent clinical need (Gao and Ma, 2019; Ishiwata et al., 2020; Soskind et al., 2020; Zhou et al., 2020). With the exception of potential rejection reactions, IFD has become a significant cause of morbidity and mortality in HSCT patients, and at present, posaconazole is often used as prophylactic therapy for prevention of IFD (Zhang et al., 2017). In clinical practice, CD children undergoing HSCT often receive multiple drugs simultaneously. However, the irrational use of drugs and harmful drug interactions could also result in serious adverse effects on the body. Nonetheless, patients with HSCT may benefit from the controlled concentrations required for tacrolimus, but if tacrolimus concentrations exceed the usual treatment ranges, it may cause a variety of serious adverse effects in patients. It has been reported that more than the upper limit of 20 ng/ml, tacrolimus may lead to toxicity (Wingard et al., 1998; Przepiorka et al., 1999), including nephrotoxicity, neurotoxicity, infection, malignancy, diabetes, and hypertension (Hoorn et al., 2011; Bentata, 2020).

Posaconazole is a well-known enzyme inhibitor implicated in numerous drug–drug interactions, among which, it includes interactions with tacrolimus (Chanoine et al., 2020), mainly due to the fact that posaconazole strongly inhibits CYP3A4 activity, which is responsible for the metabolism of a variety of medications, including tacrolimus. For example, a report by Chanoine *et al.* has described the influence of posaconazole on tacrolimus concentrations in lung transplant recipients (Chanoine et al., 2020). However, CD is an intestinal disease and may influence the *in vivo* bioavailability of tacrolimus. Furthermore, the effects of posaconazole on tacrolimus concentrations in patients with CD undergoing HSCT, and especially in children, remain unknown. The present study aimed to explore the effects of posaconazole on tacrolimus PPK in CD children undergoing HSCT. To date, the pharmacokinetic profile of tacrolimus in patients with CD subjected to HSCT remains unknown for children. The present study may be considered the first PPK study comprising a cohort of pediatric patients with CD receiving tacrolimus for HSCT.

In the present study, we established a PPK model to analyze the effects of posaconazole on tacrolimus concentrations in CD children undergoing HSCT. We found that body weight and concomitant treatment with posaconazole may be included as covariates in the final model. Other co-medications, such as glucocorticoids, mycophenolic acid, and omeprazole, were not included in the final model as covariates. Many studies had demonstrated a nonlinear relationship between drug clearance and body weight in pediatric patients, and it may be well-represented by allometric scaling using a coefficient of 0.75 for clearance and a coefficient of 1 for volume (Anderson and Holford, 2008, 2011; Chen et al., 2021). In addition, a one-compartment model was used to describe tacrolimus in the present study as all the tacrolimus concentrations evaluated were trough concentrations. Because of this clinical limitation, many studies also rely on a one-compartment model, in which body weight is included as a fixed covariable (Hao et al., 2018; Wang DD. et al., 2019; Chen et al., 2020). When it comes to Ka, there could be differences in absorption, but the current trough concentration information for tacrolimus was not enough to evaluate this variation, so we fixed Ka, which was a common protocol and one of the means to solve this clinical practical problem. This method has been used in many clinical studies. For example, Cai *et al.* reported population pharmacokinetics and dosing regimen optimization of tacrolimus in Chinese lung transplant recipients. The Ka of tacrolimus was fixed at 4.48/h (Cai et al., 2020). Ni *et al.* reported population pharmacokinetics of ciclosporin in Chinese children with aplastic anemia: effects of weight, renal function, and stanozolol administration, the Ka of ciclosporin was fixed at 0.68/h (Ni et al., 2013). Li *et al.* reported population pharmacokinetic analysis and dosing optimization of sirolimus in children with tuberous sclerosis complex; the Ka of sirolimus was fixed at 0.7521/h (Li et al., 2022).

In addition, in the present study, at the same weight, the relative value of tacrolimus clearances was 1:0.43 in children without or with posaconazole. In other words, posaconazole significantly inhibited tacrolimus clearance in CD patients undergoing HSCT. Indeed, in the present study, tacrolimus concentrations based on TDM results in children showed that posaconazole levels were, indeed, higher than those in children not receiving posaconazole. However, concentration differences were not corrected for the effects of dose and body weight. Thus, it was necessary to analyze the effects of posaconazole on tacrolimus concentrations only after adjusting for body weight and dose. We further simulated the influence of tacrolimus concentrations for different body weights (range, 5–20 kg) and at different dosage regimens (range, 0.1–0.8 mg/kg/day), and the results indicated that at the same body weight and same dose, tacrolimus steady-state concentrations in children with posaconazole were, indeed, higher than those in children without posaconazole, suggesting that posaconazole significantly increased tacrolimus concentrations in CD children undergoing HSCT, and attention should be paid to the adjustment of drug dose to prevent the occurrence of toxicity when the two drugs are combined.

For the first time, our study described the PPK profile for tacrolimus in children with CD undergoing HSCT, whereby we provided evidence that co-treatment with posaconazole significantly increased tacrolimus concentrations. Furthermore, we recommended a specific tacrolimus initial dose regimen to be used in these patients. The best probabilities of achieving the target tacrolimus concentrations were 0.6 mg/kg/day for children weighing 5–8.2 kg, 0.5 mg/kg/day for children weighing 8.2–20 kg without posaconazole, and 0.5 mg/kg/day for children weighing 5–20 kg co-treated with posaconazole. Nonetheless, independently of the administration of posaconazole, the tacrolimus concentration achieved was always highly variable in children with CD, indicating that CD disease status may affect the pharmacokinetics of tacrolimus and increase its *in vivo* variability. Therefore, TDM detection should be strengthened when children use tacrolimus to avoid toxicity in children with CD undergoing HSCT.

## Conclusion

This study, for the first time, determined the PPK for tacrolimus in children with CD undergoing HSCT. Our findings showed that cotreatment with posaconazole significantly increased tacrolimus concentrations, and we recommend a specific tacrolimus initial dose regimen to be applied to pediatric patients.

## Data Availability

The original contributions presented in the study are included in the article/Supplementary Material, further inquiries can be directed to the corresponding authors.
