# Supplementary material for: Effects of Posaconazole on Tacrolimus Population Pharmacokinetics and Initial Dose in Children With Crohn’s Disease Undergoing Hematopoietic Stem Cell Transplantation
Source: Front Pharmacol. 2022 Apr 13;13:758524. doi: 10.3389/fphar.2022.758524 (PMC9043134; doi:10.3389/fphar.2022.758524)
Supplement: Supplementary file 1 [file DataSheet1.docx]

Table S1. Demographic data between who received posaconazole and who did not.

|  | Without posaconazole | | With posaconazole | |
| --- | --- | --- | --- | --- |
|  | Median | 95% CI | Median | 95% CI |
| Age (years) | 1.60 | [0.46, 7.58] | 1.60 | [0.69, 7.58] |
| Weight (kg) | 9.40 | [5.00, 19.20] | 10.70 | [6.35, 19.70] |
| Albumin (g/L) | 34.30 | [27.50, 42.80] | 34.50 | [31.00, 42.80] |
| Alanine transaminase (IU/L) | 25.80 | [7.70, 789.40] | 45.00 | [8.00, 789.40] |
| Aspartate transaminase (IU/L) | 37.00 | [16.50, 628.20] | 33.70 | [12.50, 628.20] |
| Creatinine (μmol/L) | 17.00 | [11.00, 24.00] | 18.00 | [15.00, 28.00] |
| Urea (mmol/L) | 2.90 | [1.00, 5.20] | 3.20 | [1.50, 4.20] |
| Total protein (g/L) | 58.50 | [48.60, 71.90] | 56.70 | [52.80,71.90] |
| Total bile acid (μmol/L) | 5.20 | [1.00, 28.40] | 4.30 | [1.00, 21.60] |
| Direct bilirubin (μmol/L) | 2.30 | [1.20, 51.80] | 2.20 | [1.60, 51.80] |
| Total bilibrubin (μmol/L) | 6.60 | [2.90, 85.60] | 7.10 | [3.40, 85.60] |
| Hematocrit (%) | 30.50 | [24.10, 42.50] | 31.80 | [25.42, 42.50] |
| Hemoglobin (g/L) | 95.00 | [77.00, 147.00] | 105.00 | [80.00, 147.00] |
| Mean corpuscular hemoglobin (pg) | 24.50 | [18.30, 29.90] | 26.90 | [21.60, 28.70] |
| Mean corpuscular hemoglobin concentration (g/L) | 320.00 | [280.00, 348.00] | 336.00 | [296.00, 346.00] |

Table S2. The stepwise fashion with forward inclusion and backward elimination.

| Model | OFV | △OFV | *P*-value |
| --- | --- | --- | --- |
| **Inclusion Step** |  |  |  |
| Basic model | 1157.321 | / | / |
| CL-gender | 1156.488 | -0.833 | >0.01 |
| CL-age | 1156.230 | -1.091 | >0.01 |
| CL-albumin | 1157.229 | -0.092 | >0.01 |
| CL-alanine transaminase | 1157.188 | -0.133 | >0.01 |
| CL-aspartate transaminase | 1156.107 | -1.214 | >0.01 |
| CL-creatinine | 1156.608 | -0.713 | >0.01 |
| CL-urea | 1156.825 | -0.496 | >0.01 |
| CL-total protein | 1157.164 | -0.157 | >0.01 |
| CL-total bile acid | 1157.201 | -0.12 | >0.01 |
| CL-direct bilirubin | 1156.269 | -1.052 | >0.01 |
| CL-total bilirubin | 1156.973 | -0.348 | >0.01 |
| CL-hematocrit | 1157.214 | -0.107 | >0.01 |
| CL-hemoglobin | 1157.103 | -0.218 | >0.01 |
| CL-mean corpuscular hemoglobin | 1157.228 | -0.093 | >0.01 |
| CL-mean corpuscular hemoglobin concentration | 1157.787 | 0.466 | >0.01 |
| CL-posaconazole | 1134.398 | -22.923 | <0.01 |
| CL-glucocorticoids | 1153.560 | -3.761 | >0.01 |
| CL-omeprazole | 1156.847 | -0.474 | >0.01 |
| CL-mycophenolic acid | 1151.408 | -5.913 | >0.01 |
| **Elimination Step** |  |  |  |
| Full model (CL-posaconazole) | 1134.398 | / | / |
| Elimination of CL-posaconazole | 1157.321 | 22.923 | <0.001 |


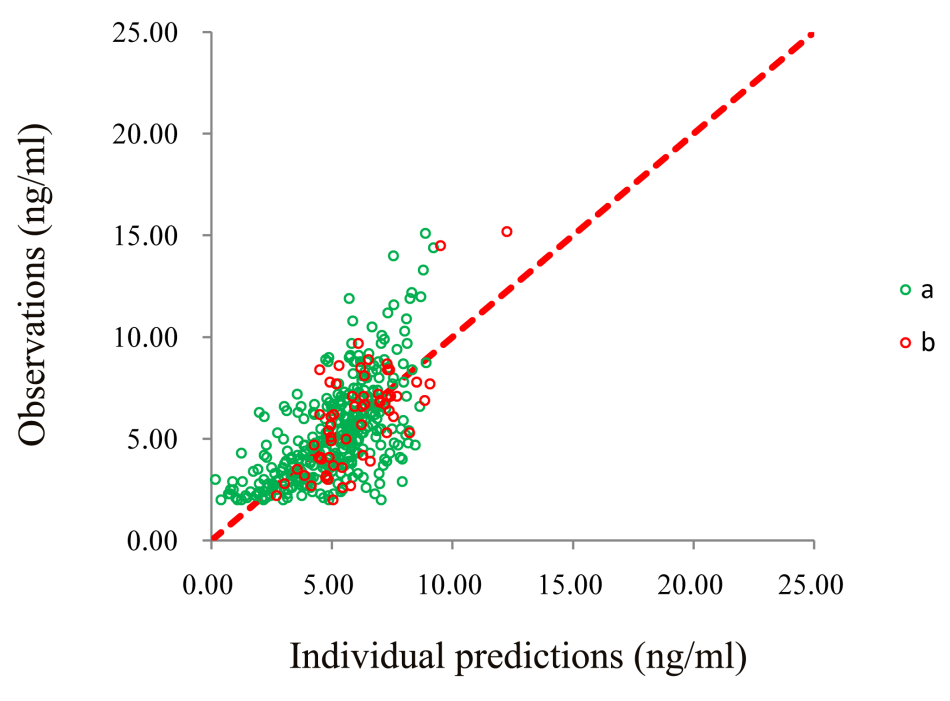


Figure S1. Observations vs. individual predictions in children with CD undergoing HSCT who received posaconazole and who did not.

a: without posaconazole. b: with posaconazole.
